# Supplementary material for: Insects, Rodents, and Pets as Reservoirs, Vectors, and Sentinels of Antimicrobial Resistance
Source: Antibiotics (Basel). 2021 Jan 12;10(1):68. doi: 10.3390/antibiotics10010068 (PMC7826649; doi:10.3390/antibiotics10010068)
Supplement: Supplementary file 1 [file antibiotics-10-00068-s001.pdf]

Table S1: Sample studies reporting the presence of antibiotic resistant bacteria in insects

Insect	Sampling site	Antibiotics/Antibiotic class	Antibiotic Resistance Genes	Organism	Methods	Country	Reference
Mealworm, Crickets	Rearing company	Tetracyclines,<br>Macrolides	tet(O, K, M, S), erm(B)	NA	Real-time PCR	Belgium and Netherlands	(Vandeweyer et al., 2019)
locusts, mealworm giant waterbugs, black ants, winged termite alates, rhino beetles, mole crickets, silkworm, and black scorpions	Retailers	Macrolides; β-lactams	Erm(B, C), tet(O, K, M, S), blaZ	NA	PCR	Netherlands and Thailand	(Milanović et al., 2016)
Small crickets (Acheta domesticus)	Retailers	tetracyclines, macrolide-lincosamide-streptogramin B (MLS_B), beta-lactams, and aminoglycosides	tet(M), tet(O), tet(K), tet(S), erm(B), erm(C), blaZ and mecA, aac(6')-Ie aph(2'')-Ia	NA	PCR	Italy	(Roncolini et al., 2019)

Cockroach	Hospital environment	Penicillins, Cephems, Aminoglycosides, macrolides. Folate inhibitors,<br>Tetracyclines, fluoroquinolones, Lincosamides, Phenicols, Ansamycins	blaZ, aacA-D, tetK, msrA, dfrA, msrA, vatB, tetM, grlA, rpoB, vatA, linA, ermA, cat1	*Staphylococcus aureus*	Culture, PCR	Iran	(Abdolmaleki et al., 2019)
Cockroaches	Household kitches	ampicillin, cephalothin, ceftriaxone, ciproflexoxacin, chloramphenicol, gentamicin, tetracycline, trimethoprim-sulfamethoxazole, ceftazidime, amikacin, nitrofurantoin, na-lidixic acid, cefalexin, cefotaxime, kanamycin, penicillin	NA	Enterobacter spp., Klebsiella<br>spp., Citrobacter spp., E. coli, Salmonella spp., Proteus spp., coagulase negative staph- ylococci, S. marcescens, Staphylococcus aureus, and Bacillus species	Culture	Iran	(Vazirianzadeh et al., 2014)
Honeybee	Bee farm	Streptomycin	strA-strB	NA	Sequencing	Norway,<br>USA	(Ludvigsen et al., 2018)

Mosquitoes	Field adult and reared larvae	beta-lactamase, Fluoroquinolones, aminoglycosides	IMI, NMC-A, OXA-18, OXA-58 Group, QnrD, OXA-60, SHV(156G), aadA1	N/A	Sequencing	USA	(Hyde et al., 2019)
House flies; cockroach	Pig farm	Tetracyclines, macrolides	tet(M), tet(O), tet(K), tet(S), erm(B)	Enterococcus spp	Culture; PCR	USA	(Ahmad et al., 2011)
True Flies	Hospital in urban areas	Penicillins, carbapenems, aminoglycosides, fluoroquinolones, quinolones, tetracyclines, phenicol, Lipopeptides, glycopeptides, Macrolides	NA	Multiple<br>Micrococcus sp., Aerococcus sp., Enterococcus spp., Streptococcus spp., Staphylococcus spp., Bacillus spp., Citrobacter spp. Klebsiella spp.<br>Leclercia spp., Pantoea spp., Enterobacter spp., Raoultella spp., Escherichia spp.	Culture	UK	(Matilla et al., 2018)

Cockroach	Households	Ampicillin, gentamicin, ciprofloxacin, ofloxacin, chloramphenicol, tetracycline, sulfamethoxa-zole/trimethoprim, penicillin, streptomycin, erythromycin, oxacillin, vancomycin, cephalothin, ceftazidime, imipenem, piperacillin, and cefoperazone	NA	Staphylococcus aureus, Enterococcus species, Pseudomonas aeruginosa, Klebsiella pneumoniae, Escherichia coli, Serratia marcescens, and Proteus species	Culture	Taiwan	(Pai et al., 2005)
Grasshopper			Erm (B, C),<br>tet(K, M, S), blaZ, aac(6')-Ie aph(2")		Culture, PCR	Netherlands and Thailand	(Osimani et al., 2017)
Flies	Cattle farm	amoxicillin/clavulanic acid; ampicillin; cefoxitin; ceftriaxone; chloramphenicol; nalidixic acid; sulfisoxazole; streptomycin; tetracycline; trimethoprim.	NA		Culture	USA	(Xu et al., 2018)

Flies	Dairy farms	Ampicillin, Cephalexin, Ceftriaxone, Ciprofloxacin, Enrofloxacin, Gentamicin, Oxacillin, Penicillin G, Sulphazotrin, Tetracycline		*Escherichia* coli, *Salmonella* spp., *Staphylococcus* spp.		Brazil	(Almeida et al., 2014)
Flies	markets, hospitals, houses/human habitations, pit latrines, stalls of local food vendors, abattoirs/slaughterhouses, and dairy farms	(tetracyclin, erythrocycline and cloxacillin) (penicillin-G and erythromycin); (streptomycin, ampicillin); (cloxacillin clindamycin) tetracycline,	NA	*Streptococcus* spp.; *E. coli*; Serratia spp. Proteus spp.; *Klebsiella* spp.	Culture	Nigeria	(Nmorsi et al., 2007)

Flies	Dairy farm	ampicillin; amoxicillin/clavulanic acid; cefalexin; ceftriaxone; cefoperazone; ceftiofur; ciprofloxacin; enrofloxacin; chloramphenicol; sulfamethoxazole/trimethoprim; gentamicin; tetracycline.	blaCTX-M, blaTEM, tetA, (int1-dfrA7-attC-qacEΔ1), dfrA7	*E. coli*	Culture + PCR	Brazil	(Alves et al., 2018)
Cockroach	Household	gentamicin; tigecycline; ciprofloxacin; cotrimoxazole; cefoxitin; cefuroxime; cefotaxime; ampicillin; aumentin; chloramphenicol; tetracycline; nitrofurantoin; aztreonam; amoxicillin/clavulanic acid, piperacillin/tazobactam; ceftaroline	*bla*$_{TEM-24}$, *bla*$_{TEM-14}$, *bla*$_{CTX-M-15/TEM-4}$, *bla*$_{CTX-M-15/TEM-1}$, *bla*$_{SHV-3, TEM-1}$, OXA-48	*E. coli*	Culture, PCR	Ghana	(Obeng-Nkrumah et al., 2018)

Mosquitoes	Residential	Doripenem, piperacillin, cefuroxime, erythromycin, ampicillin		*Staphylococcus hominis*, *Escherichia coli*, *Pantoea agglomerans,* Acinetobacter *pittii,*	Culture, MALDI TOF	Slovakia	(Hleba et al., 2017)
Blowflies and Houseflies	Farms and urban areas	Beta-lactams	*CTX-M, TEM, SHV, qnrS, LAB*	*E. coli*	Culture, PCR, Sequencing	Germany	(Wetzker et al., 2019)
Houseflies	Urban, semi-urban and rural residential areas	Clindamycin, tetracycline, penicillin, oxacillin, rifampicin, fusidic acid, trimethoprim/sulfamethoxazole, ceftriaxone, ceftazidime, aztreonam, ciprofloxacin, trimethoprim-sulfamethoxazole.	*blaCTX-M, blaTEM*	*Bacillus cereus, Enterococcus faecalis, Enterococcus faecium, Clostridioides tertium, Bacillus licheniformis, Bacillus subtilis, Enterococcus hirae, S. aureus positive*	Culture, Whole-genome sequencing	Nigeria	(Onwugamba et al., 2020)

Cockcroach	Hospital, human dwelling, restaurant	Chloramphenicol, Amoxicillin, Streptomycin, Tetracycline, Erythromycin, Gentamicin, Penicillin, Cephalothin, Sulfamethoxazole, Ciprofloxacin, Levofloxacin	*NA*	*Salmonella spp; Shigella spp; Escherichia coli; CON Staphylococcus spp; Bacillus cereus; Staphylococcus aureus; Pseudomonas aeruginosa; Klebsiella pneumoniae; Citrobacter freundii; Morganella morganii; Proteus vulgaris; Proteus mirabilis; Enterobacter cloacae; Providencia spp.*	Culture	Nigeria	(Akinjogunlaa et al., 2012)
Butterfly	Forest and densely populated human residential area	erythromycin; ciprofloxacin; norfloxacin; rifampicin.	*None*	*Enterococci*	Culture, PCR, sequencing	Brazil	(Huff et al., 2020)

Houseflies	Hospital, farm, food centre, city centre	Ampicillin; Chloramphenicol; Ciprofloxacin; Colistin; Erythromycin; Gentamicin; Imipenem; Meropenem; Streptomycin; Tetracycline.	*CTX-M, catA1,*<br>*mcr-3,*<br>*ereA*<br>*tetA, tetB,*<br>*SHV*	*E. coli*	Culture, PCR	Bangladesh	(Sobur et al., 2019)
Cockroaches	Households and restaurants	Aminoglycosides,<br>Macrolides,<br>Cephems,<br>Lincosamides,<br>Penicillins	*NA*	Staphylococcus aureus	Culture	Bangladesh	(Islam et al., 2016)
Flies	Hospital and surrounding residential suburban areas	Beta-lactams	*CTX-M-1,*<br>*TEM,*<br>*SHV,*<br>*CTX-M-9,*<br>*CTX-M-2*	*Raoultella spp.,*<br>*Kluyvera spp.,*<br>*Enterobacter spp.,*<br>*Escherichia coli,*<br>*Citrobacter spp.,*<br>*Klebsiella spp.,*<br>*Providencia spp.,*<br>*Proteus spp.,*	Culture, PCR	Ethiopia	(Tufa et al., 2020)

				Moellerella wisconsensis			
Cockroaches	Hospital and households	gentamycin; cotrimoxazole; tetracycline; chloramphenicol; amoxicillin-clavulanic acid; ciprofloxacin; nalidixic acid; ceftriaxone; ceftazidime; penicillin; erythromycin.	*NA*	*S. aureus; coagulase negative staphylococci; E. coli, Enterobacter species, Klebsiella spp.*	Culture	Ethiopia	(Moges et al., 2016)

Houseflies	Hospital, streets, abbatoir	Amoxicillin<br><br>Amoxicillin–clavulanic acid Cephaloridine<br><br>Ceftriaxone<br><br>Chloramphenicol Amikacin<br><br>Kanamycin, Norfloxacin,<br><br>Nalidixic, Doxycycline,<br><br>Trimethoprim–sulphamethoxazole	*NA*	*Escherichia coli*<br><br>*Salmonella sp. Shigella sonnei*<br><br>*Yersinia enterocolitica Edwardsiella tarda*<br><br>*Klebsiella sp.*<br><br>*Enterobacter sp. Citrobacter sp. Proteus sp.*<br><br>*Providencia sp. Serratia sp. Erwinia sp.*<br><br>*Aeromonas sp.*<br><br>*Flavobacterium sp. Pseudomonas sp. Xanthomonas sp.*<br><br>*Weeksella zoohlecum Pasteurella sp.*<br><br>*Staphylococcus sp. Streptococcus sp.*	Culture	Libya	(Rahuma et al., 2005)

Cockroach	Restaurants and cafeteria	Norfloxacin, Oxacillin, Vancomycin, Penicillin G, Clindamycin, Erythromycin, Tetracycline, Ciprofloxacin, Chloramphenicol, Cephalothin, Streptomycin	*NA*	S. aureus, B. cerus, Salmonella spp., Shigella flexneri, E. coli	Culture	Ethiopia	(Solomon et al., 2018)

Cockroaches and houseflies	Human residences	ampicillin; amoxicillin-clavulanate; piperacillin-tazobactam; cefoxitin; ceftazidime; cefepime; imipenem; ertapenem; meropenem; gentamicin; amikacin; cotrimoxazole; ciprofloxacin	*NA*	Acinetobacter lwoffi,<br>Alcaligenes spp.,<br>Citrobacter spp., Enterobacter spp.,<br>Escherichia coli, Klebsiella spp.<br>Leclercia<br>Adecarboxylata, Moellerella<br>Wisconsensis, Morganella morganii, Pasteurella spp., Proteus spp., Providencia spp., Salmonella spp., Serratia spp., Shigella dysenteriae, Yersinia enterocolitica, Staphylococcus aureus, Staphylococcus coagulase-negative Enterococcus spp.	Culture	Morocco	(Bouamama et al., 2010)

Cockroaches	Hospital	NA	*NA*	ESBL-Producing K. pneumoniae, Escherichia coli, Serratia marcescens, Enterobacter cloacae, Citrobacter freundii, Acinetobacter baumanii and Enterococcus spp	Culture	South Africa	(Cotton et al., 2000)
Cockroach	Households and restaurants	Ampicillin, piperacillin, cephalothin, chloramphenicol, tetracycline, sulfamethoxazole/trimethoprim, ceftazidime, gentamicin.	*NA*	***Pseudomonas aeruginosa, Klebsiella pneumonia, Escherichia coli, Citrobacter freundii, Enterobacter aerogenes, Proteus mirabilis***	Culture	India	(Leshan Wannigama et al., 2013)
Fly	Restaurants and farms	Amoxicillin, roxithromycin, cefaclor	*NA*	***E. coli, En. faecalis, Salmonella sp., Shigella sp.***	Culture	Australia	(Vriesekoop and Shaw, 2010)

Flies	Airport	amoxicillin, ticarcillin, cephalothin, cefuroxime, ceftazidime, piperacillin, cefotaxime, ticarcillin-clavulanate, trimethoprim-sulfamethoxazole, ciprofloxacin, gentamicin, cefepime, tobramycin, ceftazidime, cefoxitin, amikacin, netilmicin, amoxicillin-clavulanate, piperacillin-tazobactam.	*blaTEM, blaSHV acc(3)-II, aphA1 aphA2. qnrB, qnrS aac(6')- 1b.*	***Escherichia Coli, Klebsiella pneumoniae, Pseudomonas aeruginosa, Aerommas hydrophila***	Culture, PCR	China	(Liu et al., 2013)

Spiders	Garden walls and park railings	ciprofloxacin Gentamicin, amoxycillin erythromycin, chloramphenicol, tetracycline, cefoxitin, nalidixic acid, colistin	*NA*	*Pseudomonas azotoformans*,<br>*Pseudomonas peli*,<br>*Rothia mucilaginosa*,<br>*Staphylococcus capitis*<br>*Streptococcus* sp.<br>*Bacillus aerius*<br>*Bacillus altitudinis*<br>*Bacillus licheniformis*<br>*Bacillus mycoides*<br>*Bacillus mycoides*<br>*Bacillus thuringiensis*<br>*Micrococcus endophyticus*<br>*Bacillus* sp<br>*Dietzia timorensis*<br>*Micrococcus luteus*<br>*Paenibacillus mobilis*<br>*Pseudomonas putida*<br>*Rothia amarae*	Culture, PCR, Sequencing	Ireland	(Dunbar et al., 2020)

				Serratia fonticola<br><br>*Staphylococcus capitis*<br><br>*Staphylococcus edaphicus*<br><br>*Staphylococcus warneri*<br><br>*Kluyvera intermedia*<br><br>*Staphylococcus epidermidis*			

Bed gugs	Hospital	Vancomycin, ampicillin, teicoplanin, aminoglycosides	*NA*	Vancomycin Resistant Enterococci, MRSA	Culture	Canada	(Lowe and Romney, 2011)
Flies	Hospital		*Numerous genes (https://doi.org/10.1186/s13756-020-0696-y.)*	*E. coli*, *Klebsiella* spp., *Enterobacter* spp., *Aci- netobacter* spp., *P. aeruginosa*, *Citrobacter* spp., *Raoultella* spp.	Culture, MALDI_TOF, WGS	Rwanda	(Heiden et al., 2020)
Ants	Hospital	amoxicillin, amoxicillin-clav.acid, piperacillin, ticarcillin, ticarcillin-clav.acid, cephalothin, cefoxitin, cefotaxim, ceftazidim, cefepim, cefuroxim, ceftazidime, cotrimoxazol, tobramycin, gentamicin, netilmicin	*NA*	*S. aureus, A. baumannii, E.agglomerans, E. amnigenus, E. cloacae, K. oxytoca, K.pneumoniae, S. rubidae*	Culture, biochemical tests	Brazil	(Moreira et al., 2005)

Houseflies	Markets	Ampicillin,<br><br>Sulfamethoxazole-trimethoprim, Streptomycin, Tetracycline, Gentamycin, Nalidixic acid, Ceftazidime,<br><br>Chloramphenicol, Norfloxacin,<br><br>Ciprofloxacin, Cefotaxime	*blaTEM, blaSHV, blaCTX-M*	*Salmonella* spp., Enteropathogenic *E. coli*	Culture, PCR	Zambia	(Songe et al., 2017)
Houseflies	Farms	Beta-lactams, quinolones, aminoglycosides	*blaCTX-M, blaTEM, qnrS*	ESBL-producing *E. coli strains*	Culture, PCR	Spain	(Solà-Ginés et al., 2015)

References

Abdolmaleki, Z., Mashak, Z., Safarpoor Dehkordi, F., 2019. Phenotypic and genotypic characterization of antibiotic resistance in the methicillin-resistant Staphylococcus aureus strains isolated from hospital cockroaches. Antimicrob. Resist. Infect. Control 8, 1–14. doi:10.1186/s13756-019-0505-7

Ahmad, A., Ghosh, A., Schal, C., Zurek, L., 2011. Insects in confined swine operations carry a large antibiotic resistant and potentially virulent enterococcal community. BMC Microbiol. 11, 23. doi:10.1186/1471-2180-11-23

Akinjogunlaa, O.J., Odeyemib, A.T., Udoinyangc, E.P., 2012. Cockroaches (periplaneta americana and blattella germanica): reservoirs of multi drug resistant (MDR) bacteria in Uyo , Akwa Ibom State. Sci. J. Biol. Sci. 1, 19–30.

Almeida, J.L., Giuffrida, R., Andrade, R.A.P., Chaves, M.P., 2014. Muscoid Diptera as potential vectors of bacterial agents on dairy farms in the northern region of Paraná, Brazil. Semin. Ciências Agrárias 35, 3127. doi:10.5433/1679-0359.2014v35n6p3127

Alves, T. dos S., Lara, G.H.B., Maluta, R.P., Ribeiro, M.G., Leite, D. da S., 2018. Carrier flies of multidrug-resistant Escherichia coli as potential dissemination agent in dairy farm environment. Sci. Total Environ. 633, 1345–1351. doi:10.1016/j.scitotenv.2018.03.304

Bouamama, L., Sorlozano, A., Laglaoui, A., Lebbadi, M., Aarab, A., Gutierrez, J., 2010. Antibiotic resistance patterns of bacterial strains isolated from Periplaneta americana and Musca domestica in Tangier, Morocco. J. Infect. Dev. Ctries. 4, 194–201. doi:10.3855/jidc.336

Cotton, M.F., Wasserman, E., Pieper, C.H., Theron, D.C., Van Tubbergh, D., Campbell, G., Fang, F.C., Barnes, J., 2000. Invasive disease due to extended spectrum beta-lactamase-producing Klebsiella pneumoniae in a neonatal unit: The possible role of cockroaches. J. Hosp. Infect. 44, 13–17. doi:10.1053/jhin.1999.0650

Dunbar, J.P., Khan, N.A., Abberton, C.L., Brosnan, P., Murphy, J., Afoullouss, S., O'Flaherty, V., Dugon, M.M., Boyd, A., 2020. Synanthropic spiders, including the global invasive noble false widow Steatoda nobilis, are reservoirs for medically important and antibiotic resistant bacteria. Sci. Rep. 10, 1–11. doi:10.1038/s41598-020-77839-9

Heiden, S.E., Kurz, M.S.E., Bohnert, J., Bayingana, C., Ndoli, J.M., Sendegeya, A., Gahutu, J.B., Eger, E., Mockenhaupt, F.P., Schaufler, K., 2020. Flies from a tertiary hospital in Rwanda carry multidrug-resistant Gram-negative pathogens including extended-spectrum beta-lactamase-producing E. coli sequence type 131. Antimicrob. Resist. Infect. Control 9, 2–5. doi:10.1186/s13756-020-0696-y

Hleba, L., Kmeť, V., Tóth, T., Kačániová, M., 2017. Resistance in bacteria and indirect beta-lactamase detection in E. coli isolated from Culex pipiens detected by matrix-assisted laser desorption ionization time of flight mass spectrometry. J. Environ. Sci. Heal. - Part B Pestic. Food Contam. Agric. Wastes 52, 64–69. doi:10.1080/03601234.2016.1229466

Huff, R., Pereira, R.I., Pissetti, C., De Araújo, A.M., D'Azevedo, P.A., Frazzon, J., Frazzon, A.P.G., 2020. Antimicrobial resistance and genetic relationships of enterococci from siblings and non-siblings Heliconius erato phyllis caterpillars. PeerJ 2020, 1–20. doi:10.7717/peerj.8647

Hyde, J., Gorham, C., Brackney, D.E., Steven, B., 2019. Antibiotic resistant bacteria and commensal fungi are common and conserved in the mosquito microbiome. PLoS One 14, e0218907. doi:10.1371/journal.pone.0218907

Islam, A., Islam, S., Nath, A.D., Faruq, A. Al, Hassan, M.M., Islam, K., Chakma, S., Hossain, M.B., 2016. Isolation, identification and antimicrobial resistance profile of Staphylococcus aureus in Cockroaches (Periplaneta americana). J. Adv. Vet. Anim. Res. 3, 221–228.

Leshan Wannigama, D., Dwivedi, R., Zahraei-Ramazani, A., 2013. Prevalence and antibiotic resistance of gram-negative pathogenic bacteria species isolated from periplaneta americana and blattella germanica in Varanasi, India. J. Arthropod. Borne. Dis. 8, 10–20.

Liu, Y., Yang, Y., Zhao, F., Fan, X., Zhong, W., Qiao, D., Cao, Y., 2013. Multi-drug resistant gram-negative enteric bacteria isolated from flies at Chengdu Airport, China. Southeast Asian J. Trop. Med. Public Health 44, 988–996.

Lowe, C.F., Romney, M.G., 2011. Bedbugs as vectors for drug-resistant bacteria. Emerg. Infect. Dis. 17, 1132–1134. doi:10.3201/eid1706101978

Ludvigsen, J., Amdam, G. V., Rudi, K., L'Abée-Lund, T.M., 2018. Detection and Characterization of Streptomycin Resistance (strA-strB) in a Honeybee Gut Symbiont (Snodgrassella alvi) and the Associated Risk of Antibiotic Resistance Transfer. Microb. Ecol. 76, 588–591. doi:10.1007/s00248-018-1171-7

Matilla, F., Velleman, Y., Harrison, W., Nevel, M., 2018. Animal influence on water, sanitation and hygiene measures for zoonosis control at the household level: A systematic literature review. PLoS Negl. Trop. Dis. 12, 1–30. doi:10.1371/journal.pntd.0006619

Milanović, V., Osimani, A., Pasquini, M., Aquilanti, L., Garofalo, C., Taccari, M., Cardinali, F., Riolo, P., Clementi, F., 2016. Getting insight into the prevalence of antibiotic resistance genes in specimens of marketed edible insects. Int. J. Food Microbiol. 227, 22–28. doi:10.1016/j.ijfoodmicro.2016.03.018

Moges, F., Eshetie, S., Endris, M., Huruy, K., Muluye, D., Feleke, T., Silassie, F.G., Ayalew, G., Nagappan, R., 2016. Cockroaches as a Source of High Bacterial Pathogens with Multidrug Resistant Strains in Gondar Town, Ethiopia. Biomed Res. Int. 2016. doi:10.1155/2016/2825056

Moreira, D.D.O., Morais, V. de, Vieira-da-Motta, O., Campos-Farinha, A.E. de C., Tonhasca Jr., A., 2005. Ants as carriers of antibiotic-resistant bacteria in hospitals. Neotrop. Entomol. 34, 999–1006. doi:10.1590/s1519-566x2005000600017

Nmorsi, O.P.G., Agbozele, G., Ukwandu, N.C.D., 2007. Some aspects of epidemiology of filth flies: Musca domestica, Musca domestica vicina, Drosophilia melanogaster and associated bacteria pathogens in Ekpoma, Nigeria. Vector-Borne Zoonotic Dis. 7, 107–117. doi:10.1089/vbz.2006.0539

Obeng-Nkrumah, N., Labi, A.K., Blankson, H., Awuah-Mensah, G., Oduro-Mensah, D., Anum, J., Teye, J., Kwashie, S.D., Bako, E., Ayeh-Kumi, P.F., Asmah, R., 2018. Household cockroaches carry CTX-M-15, OXA-48 and NDM-1, and share beta-lactam resistance determinants with humans. bioRxiv 1–11. doi:10.1101/357079

Onwugamba, F.C., Mellmann, A., Nwaugo, V.O., Süselbeck, B., Schaumburg, F., 2020. Antimicrobial resistant and enteropathogenic bacteria in 'filth flies': a cross-sectional study from Nigeria. Sci. Rep. 10, 1–9. doi:10.1038/s41598-020-74112-x

Osimani, A., Garofalo, C., Aquilanti, L., Milanović, V., Cardinali, F., Taccari, M., Pasquini, M., Tavoletti, S., Clementi, F., 2017. Transferable antibiotic resistances in marketed edible grasshoppers (Locusta migratoria migratorioides). J. Food Sci. 82, 1184–1192. doi:10.1111/1750-3841.13700

Pai, H.H., Chen, W.C., Peng, C.F., 2005. Isolation of bacteria with antibiotic resistance from household cockroaches (Periplaneta americana and Blattella germanica). Acta Trop. 93, 259–265. doi:10.1016/j.actatropica.2004.11.006

Rahuma, N., Ghenghesh, K.S., Ben Aissa, R., Elamaari, A., 2005. Carriage by the housefly (Musca domestica) of multiple-antibiotic-resistant bacteria that are potentially pathogenic to humans, in hospital and other urban environments in Misurata, Libya. Ann. Trop. Med. Parasitol. 99, 795–802. doi:10.1179/136485905X65134

Roncolini, A., Cardinali, F., Aquilanti, L., Milanović, V., Garofalo, C., Sabbatini, R., Abaker, M.S.S., Pandolfi, M., Pasquini, M., Tavoletti, S., Clementi, F., Osimani, A., 2019. Investigating Antibiotic Resistance Genes in Marketed Ready-to-Eat Small Crickets (Acheta domesticus). J. Food Sci. 84, 3222–3232. doi:10.1111/1750-3841.14818

Sobur, A., Haque, Z.F., Sabuj, A.A.M., Ievy, S., Rahman, A.M.M.T., El Zowalaty, M.E., Rahman, T., 2019. Molecular detection of multidrug and colistin-resistant Escherichia coli isolated from house flies in various environmental settings. Future Microbiol. 14, 847–858. doi:10.2217/fmb-2019-0053

Solà-Ginés, M., González-López, J.J., Cameron-Veas, K., Piedra-Carrasco, N., Cerdà-Cuéllar, M., Migura-Garcia, L., 2015. Houseflies (Musca domestica) as vectors for extended-spectrum β-lactamase-producing Escherichia coli on Spanish broiler farms. Appl. Environ. Microbiol. 81, 3604–3611. doi:10.1128/AEM.04252-14

Solomon, F., Kibru, G., Ali, S., 2018. Multidrug-resistant pattern of food borne illness associated bacteria isolated from cockroaches in meal serving facilities, Jimma, Ethiopia. Afr. Health Sci. 18, 32–40. doi:10.4314/ahs.v18i1.6

Songe, M.M., Hang'ombe, B.M., Knight-Jones, T.J.D., Grace, D., 2017. Antimicrobial resistant enteropathogenic escherichia coli and salmonella spp. In houseflies infesting fish in food markets in Zambia. Int. J. Environ. Res. Public Health 14. doi:10.3390/ijerph14010021

Tufa, T.B., Fuchs, A., Wienemann, T., Eggers, Y., Abdissa, S., Schneider, M., Jensen, B.E.O., Bode, J.G., Pfeffer, K., Häussinger, D., Mackenzie, C.R., Orth, H.M., Feldt, T., 2020. Carriage of ESBL-producing Gram-negative bacteria by flies captured in a hospital and its suburban surroundings in Ethiopia. Antimicrob. Resist. Infect. Control 9, 1–7. doi:10.1186/s13756-020-00836-0

Vandeweyer, D., Milanović, V., Garofalo, C., Osimani, A., Clementi, F., Van Campenhout, L., Aquilanti, L., 2019. Real-time PCR detection and quantification of selected transferable antibiotic resistance genes in fresh edible insects from Belgium and the Netherlands. Int. J. Food

Microbiol. 290, 288–295. doi:10.1016/j.ijfoodmicro.2018.10.027

Vazirianzadeh, B., Dehghani, R., Mehdinejad, M., Sharififard, M., Nasirabadi, N., 2014. The First Report of Drug Resistant Bacteria Isolated from the Brown-Banded Cockroach, Supella longipalpa, in Ahvaz, South-western Iran. J. Arthropod. Borne. Dis. 8, 53–9.

Vriesekoop, F., Shaw, R., 2010. The Australian bush fly (Musca vetustissima) as a potential vector in the transmission of foodborne pathogens at outdoor eateries. Foodborne Pathog. Dis. 7, 275–279. doi:10.1089/fpd.2009.0366

Wetzker, W., Pfeifer, Y., Wolke, S., Haselbeck, A., Leistner, R., Kola, A., Gastmeier, P., Salm, F., 2019. Extended-spectrum beta-lactamase (ESBL)-producing escherichia coli isolated from flies in the urban center of Berlin, Germany. Int. J. Environ. Res. Public Health 16. doi:10.3390/ijerph16091530

Xu, Y., Tao, S., Hinkle, N., Harrison, M., Chen, J., 2018. Salmonella, including antibiotic-resistant Salmonella, from flies captured from cattle farms in Georgia, U.S.A. Sci. Total Environ. 616–617, 90–96. doi:10.1016/j.scitotenv.2017.10.324
